# Scotch Hospitals and Registration

**Published:** 1909-02-27

**Authors:** 


### Nursing Administration.

### SCOTCH HOSPITALS AND REGISTRATION.

The chief corner-stone of registration as presented in the Bill recently before the House of Lords is undoubtedly examination by a central authority. The whole nurse's career is to depend on her ability to answer in the time allotted certain questions on paper set by the examiners, and express herself readily in the further *viva voce* ordeal. In order to qualify for admission to the examination the nurse will be compelled to pass at the least three years in a hospital or hospitals of the requisite size, and offering in the eyes of the examining body the requisite advantages. But her future is to hinge on her ability to pass the new and additional examination, and the advocates of registration who have hitherto been most conspicuously before the public, regard the institution of such a final qualifying examination as the inauguration of a new era which is to regenerate the nursing world. It is interesting to note that the representative committee which is dealing with the question of State registration in Scotland take strong exception to this principle. Their objections to the legislation which has been proposed are mainly grounded on the consideration that an additional examination imposed on nurses after leaving their training schools is unnecessary and burdensome, and even likely to be harmful in its effects. The Scotch authorities advocate the examinations being left, as at present, in the hands of the training schools, under due supervision. This is the point of view which has always been consistently advocated in this paper. Sir Henry Burdett, in giving evidence before the Select Committee, May 1905, formulated a scheme for the registration of nurses under which the certificate granted by a recognised training school should entitle a nurse to registration by the General Nursing Council. The Nursing Council in this scheme was to be charged with the duty of registering or "recognising" the hospitals sufficiently well equipped to be regarded as training schools. These training centres would examine their own nurses, employing, however, outside examiners, and working under a curriculum set by the Nursing Council. They would grant, as now, their certificates of training, summing up not merely the results of a two hours' examination, but the results of three or four years' work under supervision. Then they would forward a list to the General Nursing Council as medical examining bodies now do to the Medical Council, of those who had received certificates, and these nurses would thereupon, without further anxiety or expense, and on payment of a minimum fee, receive registration certificates. The Scotch authorities are doing valuable public service in bringing forward the dangers which lurk in the institution of an outside examination for nurses. No additional examination is imposed on doctors before registration, and it is most unfair that nurses should be subjected to an ordeal of this description after passing out of their training schools with credit. It is idle to quote as precedents such examinations as those of the Central Midwives Board and of the Incorporated Society of Masseuses, held after only a few weeks' instruction, without residence. To all intents and purposes the hospital course of training for nurses is a collegiate course, and ought to rank as such. There is much reason for the fear expressed by the Scotch authorities that the institution of an extra outside examination would tend to create among nurses the impression that the main object to be aimed at in training a nurse is to enable her to pass examinations. Many admirable nurses might fail, and be stranded after creditably acquitting themselves throughout a long course of training. When a nurse fails in one paper after passing in others in her own hospital, it is possible to procure her further chances, and in the event of her being thoroughly efficient, to take into consideration her known talents in judging of her powers of expression. But the nurse who failed at her examination before the Central Board would be completly baulked in her career. So much being at stake; and time for book work being limited in hospitals, it is probable that outside coaches would spring into being, ready to teach nurses the ways of examiners, subsequent to their finishing their hospital course, and nursing would easily degenerate into a subject to be crammed. In order to prove of any value in building up a large body of registered nurses such as are required by the public, the examination cannot aim at a very high standard of theoretical knowledge, and it would obviously demand at once too much and too little. It would ask too little in being necessarily entirely divorced from any perception of a nurse's true worth in the ward, and it would demand too much in setting a nurse's career at the mercy of her memory for technical phraseology. The examinations held at present in good training schools are as nearly satisfactory as is possible for qualifying examinations to be. Held by outside examiners, they test far more than the individual talents of the nurses. They test the teaching powers of the whole establishment, together with the opportunities offered to candidates for making themselves proficient. They keep nurses and teaching staff alike up to the mark. Yet they assume no undue prominence in the training scheme. The nurse who takes highest marks is not necessarily the one selected for the coveted vacant sister's post. She understands that in granting her the certificate her work during the whole time she has been in the institution will be taken into consideration, and that the examination is merely a small part of the system which has qualified her for her future responsibilities.

Before imposing upon nurses a costly and agitating ordeal after the completion of their training due regard ought to be had to the effect of examinations in other professions. The remarks of a recent writer in the *Westminster Gazette* are only too true:

> The English boy works for an examination and despises knowledge; I do not know why, but it makes our education barren, not only in literature but in the arts; examinations as an object are the bane of our musical life, as much as of our public schools and universities.

Twenty years hence will they have become the bane of nursing education also?

